# Supplementary material for: All-at-once RNA folding with 3D motif prediction framed by evolutionary information
Source: Nat Methods. 2025 Oct 3;22(10):2094–106. doi: 10.1038/s41592-025-02833-w (PMC12510878; doi:10.1038/s41592-025-02833-w)
Supplement: Supplementary file 3 — Source code to run CaCoFold-R3D. Data and results. [file 41592_2025_2833_MOESM3_ESM.gz › supplemental_material/results/CaCoFold-R3D/CaCoFold-R3D_Rfam_vshuffle/RF01513_Afu_335.R2R.sto.pdf]

RF01513_Afu_335

5′- Y C ● ● A U G A U G A Y Y ● U C C ● G A U A G G G A C A ● C U ● A U G C ● A ● ○ ○ G C A ● A U G A U G U ● A ● C ● G G A ● A R R R R A U U U U A G U A G A U G C Y U C U U G ● U A U C U G A R ●
